# Bleeding Complications Following Paracentesis in Patients Taking Apixaban

**DOI:** 10.7759/cureus.80299

**Published:** 2025-03-09

**Authors:** Sarah Van Dorin, Andrei Schwartz, Rosarie Tudas, Kevin Sanchez, Mohammed Amarneh, Ethan Kuperman

**Affiliations:** 1 Internal Medicine, University of Iowa Hospitals and Clinics, Iowa City, USA; 2 Radiology, University of Iowa Hospitals and Clinics, Iowa City, USA

**Keywords:** abdominal paracentesis, apixaban, cirrhosis, perioperative medication management, periprocedural bleeding, therapeutic anticoagulation

## Abstract

Background

Both ascites and thrombosis are common complications of liver disease. Abdominal paracentesis to drain ascites has a low reported risk of hemorrhage, but it is unknown how exposure to direct oral anticoagulants (DOACs) such as apixaban increases this risk.

Objectives

We aim to quantify the rate of major bleeding and identify additional risk factors for bleeding in patients on apixaban undergoing paracentesis.

Methods

We performed a retrospective cohort study for all patients exposed to apixaban within seven days prior to paracentesis at a single US academic hospital between January 1, 2016, and April 1, 2022. Abstracted data included the presence or absence of hemorrhagic complications, dosing and timing of apixaban administration, and patient comorbidities.

Results

We identified 365 paracenteses in 91 unique patients. There were 20 (5.5%) reported hemorrhages, nine (2.5%) of which were plausibly related to the procedure. Four (1.1%) patients suffered fatal hemorrhage. Patients taking 10 mg twice daily of apixaban (3/8, 38%), co-prescription of apixaban with more than one additional antiplatelet or anticoagulant (3/16, 19%), apixaban taken within six hours of the procedure (6/37, 16%), and inpatient status (8/158, 5.1%) were associated with bleeding. While internal medicine residents (3/38, 7.9%) had a higher rate of hemorrhage than interventional radiologists (6/289, 2.1%), this difference was negligible when restricted to inpatients (3/38 versus 5/100).

Conclusions

The rate of bleeding after paracentesis for patients taking apixaban was much higher than historical estimates. Apixaban exposure, especially on high doses, within six hours of the procedure or on additional anticoagulant medications, significantly increases the rate of hemorrhagic complications of paracentesis.

## Introduction

Abdominal paracentesis is performed 150,000 times annually in the United States, and studies have estimated the risk of hemorrhagic complications even in the setting of thrombocytopenia and cirrhosis-related coagulopathy [1,2] at less than 1%. Based on historical data, current guidelines do not recommend withholding therapeutic anticoagulants prior to non-emergent paracentesis in most patients [3].

Recently, direct oral anticoagulants (DOACs) such as apixaban, rivaroxaban, and dabigatran have supplanted warfarin as first-line therapy for atrial fibrillation [4] and venous thromboembolism [5]. Prospective data in a pool of patients taking these medications who underwent minor procedures showed a low risk of major bleeding (0.5%). However, DOACs were continued only in 21.7% of patients, and the rest withheld therapy with or without bridging [6]. While DOACs have a safety profile comparable to vitamin K antagonists (VKAs) in cirrhosis, periprocedural data regarding paracentesis is lacking [7].

After two cases of fatal hemorrhage following abdominal paracentesis in patients on apixaban at our institution [8], we sought to quantify the rate and identify risk factors for major bleeding in patients undergoing paracentesis while on apixaban at our institution. We hypothesized that the risk of major bleeding would be above 2%.

## Materials and methods

This study, conducted at a large tertiary academic medical center, was reviewed by the University of Iowa Institutional Review Board and determined to be part of a quality improvement project, exempt from review. We identified all paracenteses performed between January 1, 2016, and April 1, 2022, in patients exposed to apixaban within seven days prior to the procedure. While apixaban is frequently held less than seven days prior to invasive surgery, this longer time interval was selected for this study due to the possibility of delayed hepatic and renal clearance in this population as well as therapeutic levels being detected in a patient with post-paracentesis bleeding at our institution more than 24 hours after last dose [8]. Inclusion criteria included age ≥18 years and a diagnostic or therapeutic paracentesis within our institution. Patients were excluded from the study if the index paracentesis occurred at an external medical center.

After screening, we divided the remaining paracenteses among all authors and independently abstracted charts for the following variables: date of paracentesis, etiology of ascites (cirrhosis, malignancy, cardiac, other), apixaban dose, last dose prior to paracentesis, antiplatelet medications, platelet count, dialysis use, creatinine, total bilirubin, international normalized ratio (INR), sodium, albumin, ascites volume, presence of encephalopathy, performing department, patient status (outpatient, inpatient, emergency department (ED)), paracentesis technique, volume of ascites withdrawn, bleeding presence, and association of bleed to paracentesis. Data were input into Research Electronic Data Capture (REDCap) [9]. If fatal or major bleeding was identified, the chart was reviewed by all authors to determine whether the bleed had a “certain,” “uncertain,” or “unlikely” relationship to the paracentesis through consensus. We defined a certain relationship of the paracentesis to bleeding as radiographic (e.g., CT, angiographic) or physical examination evidence of active bleeding near the site of paracentesis. Uncertain was defined as no radiographic evidence of an actively bleeding vessel with no other likely source of hemorrhage (e.g., no hematemesis). Unlikely was defined as a hemorrhage with a confirmed alternative source of blood loss.

The primary outcome was fatal or major bleeding occurring within 72 hours of paracentesis that had a likely or uncertain relationship with the procedure. Major bleeding was defined as a hemoglobin drop of more than 2 g/dL or transfusion receipt. Unlikely hemorrhages were excluded from this endpoint. Other parameters included other antiplatelet/anticoagulant use, Model for End-Stage Liver Disease (MELD) score, time since last apixaban dose, platelet count, and INR values. While patients with other etiologies of ascites beyond cirrhosis were included in this study, MELD and Child-Pugh scores were calculated for all patients due to the high prevalence of mild cirrhosis and the overlap between cardiac ascites and congestive hepatopathy.

Univariate analysis was used to evaluate population differences between patients with and without post-paracentesis bleeding. Chi-square testing was used to determine differences between categorical variables (e.g., patients receiving apixaban within six hours of the procedure).

## Results

We identified 365 paracenteses in 91 unique patients meeting the inclusion criteria (Table 1). Of these, 192 paracenteses were performed in the outpatient setting, 158 in inpatient units, and 15 in the emergency department. There were 21 (5.8%) paracenteses performed with confirmed last apixaban dose more than 48 hours prior to the paracentesis. Most were performed by interventional radiology (79.2%) or internal medicine (10.4%) providers. All internal medicine providers were residents. We identified 20 hemorrhages (5.5%), with nine (2.5%) (95% confidence interval (CI): 0.1-4.8) having likely or uncertain relationships to the index paracentesis (Table 2). Paracentesis of patients on apixaban had a 1.1% incidence of fatal bleeding complications (n=4).

**Table 1:** Patient characteristics (N=91) *Includes patients on prophylactic dose (n=3) and therapeutic dose (n=2) enoxaparin INR: international normalized ratio, MELD: Model for End-Stage Liver Disease

	Number (%)
Medication characteristics	
Apixaban dose	
2.5 mg bid	135 (37.0)
5 mg bid	218 (59.7)
10 mg bid	8 (2.2)
Other	4 (1.1)
Timing of last dose	
<6 hours	37 (10.1)
>6 hours	86 (23.6)
Unknown	242 (66.3)
Other anticoagulant/antiplatelet medications	
Aspirin	121 (33.2)
Clopidogrel	12 (3.3)
Enoxaparin*	5 (1.4)
Heparin	23 (6.3)
None	214 (58.6)
Patient characteristics	
Thrombocytopenia	
Yes	121 (33.2)
No	202 (55.3)
Unknown	42 (11.5)
Creatinine	
<1.8 mg/dL	159 (43.6)
≥1.8 mg/dL	176 (48.2)
Unknown	30 (8.2)
Bilirubin	
<2	225 (61.6)
≥2	60 (16.4)
Unknown	80 (21.9)
Coagulopathy	
INR < 1.7	259 (71.0)
INR ≥ 1.7	25 (6.8)
Unknown	81 (22.2)
Amount of ascites	
Slight	23 (6.3)
Moderate	340 (93.2)
Unknown	2 (0.5)
Encephalopathy	
Grade 1-2	97 (26.6)
Grade 3-4	8 (2.2)
No encephalopathy	260 (71.2)
MELD score	
Mean	16.6 (4.5)
Standard deviation	6.6 (1.8)
Unknown	104 (28.5)
Child-Pugh class	
A	1 (0.3)
B	180 (49.3)
C	78 (21.4)
Unknown	106 (29.0)
Patient status	
Emergency department	15 (4.1)
Inpatient	158 (43.3)
Outpatient	192 (52.6)
Etiology of ascites	
Cardiac	98 (26.8)
Cirrhosis	140 (38.4)
Malignancy	52 (14.2)
Combination or other	75 (20.5)
Paracentesis characteristics	
Performing department	
Internal medicine	38 (10.4)
Interventional radiology	289 (79.2)
Other	38 (10.4)
Fluid withdrawn (L)	
<0.5	39 (10.7)
0.5-1.99	29 (7.9)
2-5	120 (32.9)
>5	174 (47.7)
Not documented	3 (0.8)

**Table 2:** Characteristics of patients with bleeding likely or uncertainly related to index paracentesis (n=9) MELD: Model for End-Stage Liver Disease

Patient	Apixaban dose	Timing of dose	Other antiplatelet/anticoagulant agents	Etiology of ascites	MELD	Child-Pugh class	Patient status	Performing department	Bleeding severity	Relationship to paracentesis
1	5cmg bid	<6 hours	None	Cardiac	16	7	Inpatient	Interventional radiology	Major	Likely
2	10 mg bid	<6 hours	Aspirin	Cirrhosis	16	11	Inpatient	Interventional radiology	Major	Uncertain
3	10 mg bid	>6 hours	None	Cirrhosis	15	9	Inpatient	Internal medicine	Major	Uncertain
4	2.5 mg bid	Unknown	Aspirin, heparin	Cardiac	21	7	Inpatient	Internal medicine	Major	Uncertain
5	5 mg bid	<6 hours	Aspirin, clopidogrel	Cardiac	21	8	Inpatient	Interventional radiology	Major	Likely
6	10 mg bid	<6 hours	None	Cirrhosis	23	9	Inpatient	Internal medicine	Fatal	Likely
7	5 mg bid	<6 hours	Aspirin	Cirrhosis	22	10	Outpatient	Interventional radiology	Fatal	Likely
8	2.5 mg bid	>6 hours	Aspirin, clopidogrel	Combination	21	9	Inpatient	Interventional radiology	Fatal	Likely
9	2.5 mg bid	<6 hours	None	Malignancy	15	9	Inpatient	Interventional radiology	Fatal	Uncertain

A univariate analysis of the odds ratios for bleeding for multiple risk factors is presented in Table 3. Co-prescription of apixaban with more than one additional anticoagulant or antiplatelet agent (e.g., aspirin, clopidogrel, or enoxaparin) (n=16) had the strongest association with bleeding risk, with 19% of these patients experiencing hemorrhage. Higher rates of bleeding complications were observed for apixaban dosing within six hours of paracentesis (16%, 6/37) when compared to those with a longer preprocedural hold (2.3%, 2/86), but there was a high rate of missing data (n=242). Inpatients were more likely to experience bleeding than outpatients (8/158 versus 1/192) (P=0.008). Paracentesis performed by internal medicine residents (7.9% 3/38) compared to an interventional radiologist (2.1%, 6/289) (P=0.039 for difference) was also associated with bleeding. However, on post hoc analysis, this difference was negligible when restricting the comparison to the inpatient subgroup (3/38 versus 5/100) (P=0.516), suggesting that internal medicine providers may have been performing the procedure in a higher-risk population. Unsurprisingly, patients on the higher 10 mg twice daily dose of apixaban had a higher rate of major bleeding than those on the standard 5 mg twice daily dose (3/8 versus 3/218) (P<0.001). Potentially due to the small sample size, there was no statistically significant association with coagulopathy, MELD score, Child-Pugh category, platelet count, or creatinine with hemorrhage.

**Table 3:** OR for major or fatal bleeding complications after paracentesis *P<0.05 by Chi-square test **P<0.001 by Chi-square test OR: odds ratio, CI: confidence interval, INR: international normalized ratio, MELD: Model for End-Stage Liver Disease

Risk factor	OR (95% CI)	P-value
Medication characteristics		
Apixaban dose		
2.5 mg bid	0.6 (.1-4.2)	0.55
5 mg bid	Reference	N/A
10 mg bid	26.4 (4.2-165.0)**	<0.001
Other	No bleeding reported	N/A
Timing of last apixaban dose		
<6 hours	8.1 (1.6-42.4)*	0.004
>6 hours	Reference	N/A
Unknown	0.2 (0.0-1.6)	0.106
Other anticoagulant and antiplatelet medications		
Single agent	0.8 (0.1-4.2)	0.756
Multiple agents	12.1 (2.5-59.9)**	<0.001
None	Reference	N/A
Patient characteristics		
Thrombocytopenia		
Yes	0.5 (0.1-2.3)	0.338
No	Reference	N/A
Unknown	No bleeding reported	N/A
Creatinine		
<1.8 mg/dL	Reference	N/A
>1.8 mg/dL	3.3 (0.7-15.9)	0.124
Unknown	No bleeding reported	N/A
Bilirubin		
<2 mg/dL	Reference	N/A
>2 mg/dL	1.1 (0.2-5.3)	0.930
Unknown	No bleeding reported	N/A
Coagulopathy		
INR < 1.7	Reference	N/A
INR > 1.7	3.1 (0.6-16.0)	0.149
Unknown	No bleeding reported	N/A
Encephalopathy		
Absent	Reference	N/A
Present	1.2 (0.4-4.2)	0.722
MELD score		
<21	Reference	N/A
>21	0.8 (0.2-3.3)	0.772
Unknown	No bleeding reported	N/A
Child-Pugh class		
A	No bleeding reported	N/A
B	Reference	N/A
C	0.7 (0.1-3.2)	0.594
Unknown	No bleeding reported	N/A
Patient location		
Outpatient	Reference	N/A
Emergency department	No bleeding reported	N/A
Inpatient	10.2 (1.3-82.3)*	0.008
Etiology of ascites		
Cardiac	1.1 (0.2-4.9)	0.927
Cirrhosis	Reference	N/A
Malignancy	0.7 (0.1-6.1)	0.718
Combination or other	0.5 (0.1-4.2)	0.480
Paracentesis characteristics		
Performing department (all patients)		
Internal medicine	4.0 (1.0-16.9)*	0.039
Radiology	Reference	N/A
Other	No bleeding reported	N/A
Performing department (inpatient only)		
Internal medicine	1.6 (0.4-7.2)	0.516
Radiology	Reference	N/A
Other	No bleeding reported	N/A
Fluid withdrawn		
<0.5 L	Reference	N/A
0.5-5 L	0.5 (0.1-2.9)	0.445
>5 L	0.3 (0.1-2.0)	0.227
Not documented	No bleeding reported	N/A

## Discussion

In this retrospective cohort study, the risk of major bleeding after paracentesis for patients who received apixaban was significantly higher than in previous reports. Co-prescription of apixaban with another antiplatelet/anticoagulant agent and lack of withholding apixaban prior to procedure also increased bleeding risk.

A recent study found that uninterrupted periprocedural anticoagulation did not increase hemorrhage after paracentesis, although only 18 patients were treated with a DOAC [10]. While there is a lack of demonstrated association with post-paracentesis bleeding and anticoagulation, previous studies evaluating the risk of all bleeding for patients on aspirin, clopidogrel, and VKAs have found a threefold overall higher risk for patients on triple therapy when compared to patients on monotherapy [11,12]. Few studies have evaluated the procedural risk of bleeding for patients on triple therapy or if bleeding risk changes if the patient is on a DOAC instead of a VKA. Per the Society of Interventional Radiology (SIR), dual antiplatelet therapy (DAPT) can be continued for procedures with a low bleeding risk (such as paracentesis) [3]. Similarly, SIR recommends the continuation of DOACs if both the procedure and the patient have a low risk of bleeding. If the patient is at high risk, it is recommended that the anticoagulant be withheld.

According to the American College of Cardiology’s Decision Pathway for Periprocedural Anticoagulation, DOACs are recommended to be withheld if the procedure is considered low, uncertain, intermediate, or high risk of bleed [13]. Patients may continue DOACs for procedures with no clinically significant risk of bleed, but it is recommended to perform the procedure when the DOAC level is at a trough. In the Appendix of that document, paracentesis is listed as having equivalent risk of bleeding to an arterial puncture or skin biopsy, procedures routinely done on therapeutic anticoagulation [11,12].

The Perioperative Anticoagulation Use for Surgery Evaluation (PAUSE) trial evaluated the impact of withholding DOACs before elective procedures and reported that 30-day postoperative rates of major bleeding were <2% and the risk of stroke was <1% when anticoagulants were withheld prior to procedure [14]. In our study, a majority (6/9) of associated hemorrhages occurred when apixaban was given <6 hours prior to paracentesis. The maximum blood concentration for apixaban is reached 3-4 hours after oral administration, with a half-life of around 12 hours [15]. As major prospective studies on apixaban [16,17] have excluded patients with hepatic impairment (e.g., total bilirubin 1.5 times upper limit of normal), there is insufficient data to determine further dosing recommendations for patients with advanced liver disease. However, two studies reported no significant increase in apixaban AUC in Child-Pugh classes A and B liver disease [18,19].

Patients on a combination of oral anticoagulation with dual antiplatelet therapy (“triple therapy”) have increasingly been recognized as having a prohibitively high long-term risk of bleeding [20]. Current consensus guidelines recommend limiting triple therapy after percutaneous coronary intervention to one month and only in patients with “acceptable bleeding risk” [21]. Patients on multiple agents that impair coagulation may require a more extended period of withholding anticoagulation preoperatively or additional precautions taken to prevent or monitor hemorrhage (e.g., ultrasound visualization during catheter insertion).

In our study, paracenteses performed by internal medicine residents had a higher risk of major bleeding compared to those performed by interventional radiology. Many factors likely played a role, including acuity of patients, training of the performing provider, and provider experience. The ultrasound curriculum developed for internal medicine residents at our institution emphasizes diagnostic procedures [22] but lacks formal paracentesis ultrasound technique training (evaluating for abdominal vessels, identifying fluid pockets, and direct visualization of catheter insertion), which is recommended for paracentesis by the Society of Hospital Medicine [23]. Internal medicine residents attend simulation sessions at the beginning of the academic year for multiple procedures (paracentesis, lumbar puncture, and others), but this does not include procedure-specific ultrasound training. The number of paracenteses performed also varies greatly between internal medicine residents based on procedural interest. Bedside faculty supervision is optional, and faculty are not formally evaluated for competence with the procedure. Potential changes to decrease the risk of hemorrhage include increasing paracentesis training for internal medicine residents and faculty (including dedicated abdominal ultrasound skills), increasing procedural exposure (reintroducing procedural service), or uniform supervision by qualified staff.

Our study has several limitations, including a small sample size (91 unique patients) at a single institution and insufficient event numbers for multivariate analysis or interaction between risk factors. While most patients had some degree of liver injury, less than 40% had cirrhosis as the sole documented cause of ascites and only 20% had documented Child-Pugh class C liver disease, which may continue to limit our understanding of the safety of apixaban in patients with advanced cirrhosis. The inclusion of patients with cirrhosis from other etiologies may explain why advanced liver disease did not correlate with higher bleeding risk. There was also a high rate of missing data for the timing of the last apixaban dose and laboratory values in patients undergoing outpatient procedures. Finally, other risk factors for bleeding such as repeated use of the same paracentesis site could not be abstracted reliably from this data set and were not included in this analysis.

## Conclusions

Observed hemorrhagic complications in our study cohort were much higher than in previous reports in patients on apixaban receiving paracentesis. This contrasts with previous reports that patients with coagulopathy or thrombocytopenia secondary to cirrhosis are not at increased risk of bleeding. Patients on apixaban 10 mg twice daily or combined with dual antiplatelet therapy may have substantial risk. When feasible, the procedure should be delayed more than six hours after prior apixaban dose. In patients with additional risk factors, such as receipt of antiplatelet agents, the risk of bleeding may outweigh the risks of thrombosis and justify longer apixaban withholding. Future multicenter or prospective trials may be necessary to further define best practices for this high-risk population.
